# An In-Bore Audio-Visual Respiratory Coaching Projector for Ring Gantry Systems

**DOI:** 10.7759/cureus.70653

**Published:** 2024-10-01

**Authors:** Masao Tanooka, Keisuke Sano, Wataru Okada

**Affiliations:** 1 Department of Radiotherapy, Takarazuka City Hospital, Takarazuka, JPN

**Keywords:** 4dct, audio-visual, in-bore projector, radiotherapy, respiratory coaching, ring gantry

## Abstract

We have proposed an in-bore audio-visual respiratory coaching projector for use with ring gantry linacs and four-dimensional computed tomography (4D CT) systems. A respiratory coaching application was installed on a commercially available mobile projector that includes a computer. The projector was placed on a patient couch, with the projected movie displayed on the inner wall of the bore. The workflow was tested using a ring gantry linac as well as a 4D CT unit, and the results demonstrated the feasibility and practicality of the proposed approach.

## Introduction

It is known that audio-visual coaching represents an efficacious method for stabilizing patient respiration [1]. Okada et al. asserted that audio coaching resulted in more stable respiratory motion during stereotactic liver radiotherapy [2]. Kim et al. proposed a visual guidance device for an Ethos ring gantry linac, utilizing a standard-size projector positioned outside the linac bore, with the projected video displayed on the inner wall of the bore [3]; however, it may not be always the case that a standard-size projector represents the optimal choice for routine use. The purpose of this paper was to propose an in-bore audio-visual respiratory coaching projector and assess its feasibility and practicality.

## Technical report

Materials and methods

A commercially available mobile projector (X-03, FunLogy, Chiba, Japan) was utilized. The mobile projector is equipped with a computer that runs on the Android operating system (OS) and a speaker. In a related development, Anzai Medical (Tokyo, Japan) had previously created a respiratory coaching and monitoring unit, ABLE (Anzai Breath Learning Equipment), as a part of their respiratory synchronizer system, i.e., AZ-733VI (Anzai Medical). Following discussions with Anzai Medical, they decided to install the ABLE application on the mobile projector. The new respiratory coaching-only standalone unit was designated "ABLE AP," and the preliminary prototype was provided for our assessment. In the ABLE AP, no respiratory monitoring functionality was provided, and therefore it is a standalone coaching-only device.

In this study, no patients were included and the participants of the tests were the authors of this article, where a ring gantry linac, Radixact (Accuray, Madison, Wisconsin), and a four-dimensional computed tomography (4D CT) machine, Aquilion LB (Canon Medical Systems, Tochigi, Japan), were employed.

Results

**Figure 1:**
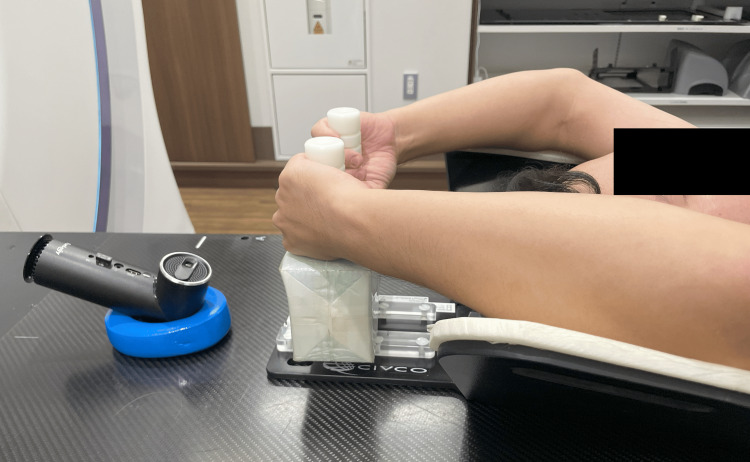
The projector placed on a patient couch. The projector is positioned on a patient couch at an oblique angle to project the respiratory coaching image on the inner wall of the bore of the ring gantry linac.

Figure 1 depicts the mobile projector positioned on a patient couch. The projector was situated beyond the volunteer's head at an oblique angle, projecting the respiratory coaching movie onto the inner wall of the bore, right above the eyes, on the ring gantry linac.

Video 1 illustrates the procedure for setting up the respiratory coaching application. Prior to utilization, the configuration screen was displayed on the ceiling of the linac room, wherein two distinct transient times from peak expiration to peak inspiration and vice versa could be configured. For easy configuration on the screen, a USB mouse was connected to the projector.

**Video 1:** Setting up the respiratory coaching application in the projector. Prior to the use, the setup screen is displayed on the ceiling of the linac room.

Video 2 shows an in-bore respiratory coaching video projected onto the inner wall of the linac bore. The video shows back-and-force motion of a ball with a voice of “breathe in” and “breathe out” coming from the built-in speaker inside the projector.

**Video 2:** In-bore respiratory coaching video projected on the inner wall of the linac bore.

Video 3 indicates respiratory coaching for a 4D CT imager. Due to its shorter bore length, the projected video was displayed on the surface or the inner wall of the bore, depending on the projector and the patient positions.

**Video 3:** Respiratory coaching for a 4D CT unit. The projected image is displayed on the surface of the CT device. 4D CT: four-dimensional computed tomography.

## Discussion

Our findings indicate that it was feasible to place the mobile projector on a patient couch and that it was practical to project the respiratory coaching movie onto an inner wall of the bore on the ring gantry linac, such as the Radixact. Compared to Kim’s standard-size projector approach [3], no extended screen may be required because we have more degrees of freedom to place the mobile projector on the patient couch. Our mobile projector has an operating system and a CPU, so the whole coaching system is integrated into a compact projector, minimizing the impact on the technologist's workflow. The authors believe this to be a significant contribution to ensuring safe and secure treatment.

Additionally, it was reported that audio coaching resulted in more stable respiratory motion during stereotactic liver radiotherapy [2]. It is expected that the combination of visual and audio coaching may further facilitate stability of the patient respiration during stereotactic liver radiotherapy, which will be the subject of our subsequent investigation.

We have only tested feasibility and practicality through the Radixact. However, other ring gantry linacs, including Accuray’s Tomotherapy, Varian’s Ethos/Halcyon, and Hitachi’s OXRAY, are likely to work in the same way. The principal benefit of this standalone projector is that it can be employed with any ring gantry linac system that incorporates any respiratory management solution, thereby facilitating respiratory stability.

Our findings also suggest that a 4D CT unit may have the potential to work with the proposed approach. However, further studies are needed to optimize the position and orientation of the mobile projector because the CT unit has a shorter bore length. To the authors’ knowledge, this is the first report that demonstrates the possibility of an in-bore audio-visual respiratory coaching projector for stable breathing during 4D CT image acquisition.

The optimal oblique angle of the projector may depend on the position of thoracoabdominal tumors and patient heights, which could be a topic for further investigation in a clinical setting.

## Conclusions

We have successfully demonstrated the feasibility and practicality of the audio-visual mobile projector for daily use. It is anticipated that the in-bore projector may facilitate more stable patient respiration during radiotherapy by a ring gantry linac. Furthermore, the in-bore audio-visual mobile projector may be employed for 4D CT image acquisition, thereby possibly enabling more accurate 4D image reconstructions.

## References

[1] Goossens S, Senny F, Lee JA, Janssens G, Geets X (2014). Assessment of tumor motion reproducibility with audio-visual coaching through successive 4D CT sessions. J Appl Clin Med Phys.

[2] Okada W, Doi H, Tanooka M (2021). A first report of tumour-tracking radiotherapy with helical tomotherapy for lung and liver tumours: a double case report. SAGE Open Med Case Rep.

[3] Kim T, Ji Z, Lewis B (2022). Visually guided respiratory motion management for Ethos adaptive radiotherapy. J Appl Clin Med Phys.

